# The Epidemiology of HIV and HSV-2 Infections among Women Participating in Microbicide and Vaccine Feasibility Studies in Northern Tanzania

**DOI:** 10.1371/journal.pone.0068825

**Published:** 2013-07-18

**Authors:** Saidi H. Kapiga, Fiona M. Ewings, Tony Ao, Joseph Chilongani, Aika Mongi, Kathy Baisley, Suzanna Francis, Aura Andreasen, Ramadhan Hashim, Deborah Watson-Jones, John Changalucha, Richard Hayes

**Affiliations:** 1 London School of Hygiene and Tropical Medicine, London, United Kingdom; 2 Mwanza Intervention Trials Unit, Mwanza, Tanzania; 3 National Institute for Medical Research, Mwanza, Tanzania; Infectious Disease Service, United States of America

## Abstract

**Objectives:**

To prepare for future HIV prevention trials, we conducted prospective cohort studies among women working in food and recreational facilities in northern Tanzania. We examined the prevalence and incidence of HIV and HSV-2, and associated risk factors.

**Methods:**

Women aged 18–44 years working in food and recreational facilities were screened to determine their eligibility for the studies. Between 2008–2010, HIV-negative women were enrolled and followed for 12 months. At enrolment and 3-monthly, we collected socio-demographic and behavioural data, and performed clinical examinations for collection of biological specimens that were tested for reproductive tract infections. Risk factors for HIV and HSV-2 incidence were investigated using Poisson regression models.

**Results:**

We screened 2,229 and enrolled 1,378 women. The median age was 27 years (interquartile range, IQR 22, 33), and median duration working at current facility was 2 years. The prevalences of HIV at screening and HSV-2 at enrolment were 16% and 67%, respectively. Attendance at the 12-month visit was 86%. HIV and HSV-2 incidence rates were 3.7 (95% confidence interval, CI: 2.8,5.1) and 28.6 (95% CI: 23.5,35.0)/100 person-years, respectively. Women who were separated, divorced, or widowed were at increased risk of HIV (adjusted incidence rate ratio, aRR = 6.63; 95% CI: 1.97,22.2) and HSV-2 (aRR = 2.00; 95% CI: 1.15,3.47) compared with married women. Women reporting ≥3 partners in the past 3 months were at higher HIV risk compared with women with 0–1 partner (aRR = 4.75; 95% CI: 2.10,10.8), while those who had reached secondary education or above were at lower risk of HSV-2 compared with women with incomplete primary education (aRR = 0.42; 95% CI: 0.22,0.82).

**Conclusions:**

HIV and HSV-2 rates remain substantially higher in this cohort than in the general population, indicating urgent need for effective interventions. These studies demonstrate the feasibility of conducting trials to test new interventions in this highly-mobile population.

## Introduction

HIV infection remains a major public health problem despite evidence of declining incidence in many parts of the world and increasing access to antiretroviral therapy. Tanzania is one of the countries within sub-Saharan Africa severely affected by the HIV epidemic, with an estimated 1.5 million people living with HIV at the end of 2010 [1]. Based on national surveys, the prevalence of HIV among the general adult population was estimated to be 7.0% in 2003–4 [2] and 5.7% in 2007–8 [3].

In Tanzania, HIV prevalence is generally higher among women than men [2], [3], indicating that the impact of the epidemic is not uniform. In particular, women working in hotels, restaurants, bars and other food and recreational facilities have substantially higher HIV prevalence and incidence than women in the general adult population [4]–[7]. Although several factors have been postulated to account for the increased HIV vulnerability among these women, including being involved in complex sexual networks with multiple partners and exchange of sex for gifts or money, more studies are needed to investigate new strategies to help reduce the burden of HIV in this population.

Most current HIV prevention methods require the consent (as well as some action or behaviour change) of the male partner. Correct and consistent use of latex condoms is one proven method of preventing HIV transmission; however, condoms are widely regarded as inadequate prevention options for women, because many women are unable to negotiate condom use with their partners [8]. Male circumcision is another proven HIV prevention method but there is no evidence that this is effective in reducing transmission from men to women [9]. Thus, the search for new prevention methods, such as microbicides and vaccines, remains a major global public health priority.

In order to prepare for future HIV prevention trials, we conducted two prospective cohort studies among women working in hotels, restaurants, bars and other food and recreational facilities in northern Tanzania. The objectives were to assess feasibility, retention and appropriateness of this population for future trials. In this report, we describe the prevalence and incidence of HIV and herpes simplex virus type 2 (HSV-2), and examine their risk factors. We examined risk factors for HSV-2, in addition to HIV, because HSV-2 is a common sexually transmitted infection (STI) known to be associated with increased risk of HIV acquisition [10].

## Methods

### Ethics Statement

The studies were approved by the Ethics Committees of the National Institute for Medical Research (NIMR), Kilimanjaro Christian Medical Centre (KCMC), and London School of Hygiene and Tropical Medicine. All participants received detailed information about the study to ensure that they understood why the study was being carried out, what the study involved and the involvement of the three collaborating research institutions. Furthermore, they were informed that participation in the study was voluntary and written informed consent (signature or witnessed thumbprint) was obtained prior to screening and enrolment. Participants’ confidentiality was ensured by excluding personal identification (such as names) from forms used to collect study information and by storing study documents and samples in a secure location. Study staff were trained on issues of confidentiality, good clinical practice, research ethics and protection of human subjects. At the end of the study, participants were invited to dissemination meetings where the main findings were presented and discussed.

### Study Populations and Recruitment

Between 2008–2010, we recruited women working in hotels, restaurants, bars, guesthouses or shops selling traditionally-brewed beer, or food-sellers at makeshift facilities (*mamalishe*) in the northern Tanzanian towns of Geita, Kahama, Shinyanga (for the microbicides-preparedness study) and Moshi (for the vaccines-preparedness study). Three of these towns have large-scale gold (Geita and Kahama) and diamond (Shinyanga) mines which attract a large number of men seeking employment. Shinyanga, Kahama and Moshi are also along major highways connecting northern Tanzania with the capital city of Dar es Salaam and other countries in the East African region, while Moshi, on the slopes of Mount Kilimanjaro, is also a major destination for international tourism and has a high number of hotels and guesthouses. These factors may contribute to increased risk of HIV among women residing in these towns.

In each town, we determined the number of facilities located in each local ward, the governmental administrative unit responsible for a defined geographical area. Enrolment began in the ward with the largest number of facilities and progressed to the ward with the next largest number of facilities until the study sample size was reached. The target sample size was 1000 women for the microbicides-preparedness cohort and 500 for the vaccines-preparedness cohort. In each selected local administrative ward, study staff met with local leaders, facility owners, facility managers, and health officials to inform them about the study objectives before commencing data collection. Women were eligible for enrolment if they were aged 18–44 years, willing to undergo HIV testing and to receive results, and not planning to move away for the duration of the study. We enrolled HIV-negative women, although some HIV-positive women were enrolled at the Moshi site for a separate sub-study to investigate the molecular epidemiology of HIV.

### Study Procedures

In each participating food and recreational facility, we invited all women employed at the facility to visit the study clinic located within (for Shinyanga, Kahama and Moshi towns) or near (for Geita town) an existing public hospital for more detailed information about the study and an eligibility assessment as part of the screening process. During the screening visit, women underwent a brief interview to collect limited information about demographic variables, behavioural risk factors for STIs and other information to determine their study eligibility. HIV rapid testing was performed, with pre- and post-test counselling, and blood samples were collected for those with concordant positive or discordant test results for confirmatory testing. However, in Moshi, those determined ineligible for other reasons were not tested.

Women confirmed to be HIV-negative and fulfilling other inclusion criteria were invited to come to an enrolment visit within 4 weeks. At enrolment, structured face-to-face interviews were conducted to obtain information about socio-demographic characteristics, employment, reproductive history, sexual behaviour and work mobility. Information about alcohol use, including screening for problem drinking, was obtained using CAGE [11] and Alcohol Use Disorders Identification Test (AUDIT) [12] questionnaires. Samples of 5–10 ml of whole blood were collected for detection of syphilis, HSV-2 and HIV infections. A clinical examination was performed and genital samples for detection of other STIs and other genital infections were collected.

All women enrolled in the study were scheduled to return to the clinic every three months for 12 months. During each visit, similar interviews, clinical examinations and blood and genital sample collection were performed as at enrolment.

Blood and genital samples were transported to either a laboratory at NIMR in Mwanza city (microbicides-preparedness cohort) or KCMC in Moshi (vaccines-preparedness cohort) for further processing. Study participants with positive laboratory results for curable STIs (*Neisseria gonorrhoeae*, *Chlamydia trachomatis, Trichomonas vaginalis* or syphilis) or HIV were asked to return to the research clinic as soon as possible for post-test counseling and treatment as appropriate. Women with STI-related symptoms or laboratory-confirmed infections at any time received treatment at no cost, in accordance with the Tanzanian Ministry of Health guidelines.

### Laboratory Methods

Testing for infections was performed according to standard operating procedures in each of the NIMR and KCMC laboratories. At all sites, HIV rapid testing was performed at screening in parallel using SD Bioline HIV-1/2 3.0 (Standard Diagnostics, Inc., Korea) and Determine HIV-1/2 (Alere Medical, Co., Ltd, Japan) tests. If the rapid tests were positive or discordant, HIV infection was confirmed in the respective laboratories using either third generation Murex HIV 1.2.O (Abbott UK, Dartford, Kent, England) and Vironostika HIV Uniform II plus O (bioMérieux Bv, The Netherlands) enzyme-linked immunosorbent assays (ELISAs; microbicides-preparedness cohort), or only Vironostika HIV Uniform II plus O ELISA (vaccines-preparedness cohort). In the microbicides-preparedness cohort, samples discrepant or indeterminate on ELISA were tested for P24 Antigen (Genetics Systems HIV-1 Ag EIA, Bio-rad Laboratories, Marnes-la-Coquette, France) and if positive were classified as HIV-positive. Samples negative for P24 antigen were tested by Western Blot (INNO-LIA, HIV I/II score, Innogenetics NV, Gent, Belgium). At enrolment and follow-up visits, HIV testing was done using ELISAs as per the screening algorithm. In the vaccines-preparedness cohort, the HIV testing algorithm used at screening was applied at enrolment and throughout the follow-up period.

In the microbicides-preparedness cohort, syphilis was determined by the Immutrep Rapid Plasma Reagin (RPR) card test (Omega Diagnostics, Alva, Scotland) and the *Treponema pallidum* particle hemagglutination assay (SERODIA TPPA, Fujirebio Inc., Tokyo, Japan). Previous syphilis infection was diagnosed if TPPA-positive but RPR-negative, and active syphilis if TPPA–positive and RPR-positive. Based on RPR titre, active syphilis was categorised as low (<1∶8) or high (≥1∶8) titre. Syphilis results were not available for the vaccines-preparedness cohort. HSV-2 was detected using either type-specific IgG ELISA (Kalon Biologicals Ltd., Guildford, UK; microbicides-preparedness cohort) or Herpes Select™ 2 ELISA IgG assay (Focus Diagnostics, Cypress, CA, USA; vaccines-preparedness cohort). Endocervical swabs were collected for *N. gonorrhoeae* and *C. trachomatis* detection by Amplicor PCR (Roche Diagnostics, Branchburg, NJ, USA). All positive tests for *N. gonorrhoeae* were confirmed using specific primers to the -16S DNA coding region in PCR in-house assays (Sigma-Aldrich, UK) [13]. In the microbicides-preparedness cohort, a vaginal swab was used to inoculate a culture (InPouch TV, Biomed Diagnostics, San Jose, CA, USA), read by light microscopy at 72 hours after incubation at 36–37°C, and a second vaginal swab was rolled onto a slide, heat-affixed, gram-stained and Nugent-scored for bacterial vaginosis [14]; these tests were not performed in the vaccines-preparedness study. All laboratory tests were performed at enrolment and every 3 months, except for *N. gonorrhoeae*, *C. trachomatis* and *T. vaginalis*, which were measured at enrolment and months 6 and 12 only.

### Statistical Methods

Questionnaires were reviewed for completeness and consistency at the research clinics and data were double-entered using OpenClinica (Akaza Research, Waltham, MA, USA) or DMSys software (SigmaSoft International, Chicago, IL, USA). Data were analysed using Stata version 11 (StataCorp, College Station, TX, USA).

Attendance rates by scheduled visit were determined as the number of women who attended a visit divided by the number of women enrolled. Rates of enrolment (among those screened) and attendance (of those enrolled) were compared between the cohorts using Χ^2^ tests.

Factors associated with HIV prevalence at screening, and HSV-2 prevalence at enrolment, were examined using logistic regression. HIV and HSV-2 incidence during follow-up was calculated among women who were negative for each infection at enrolment, as the number of seroconversions divided by the total person-years (PYRs) of observation. For each infection, the date of seroconversion was assumed to be halfway between the last negative and first positive results. Follow-up was included to the earliest of seroconversion date or date last seen. The associations between potential risk factors and seroconversion were summarised by using incidence rate ratios (RR) from Poisson regression models. Statistical significance of the models was assessed using likelihood-ratio tests.

All models included age and town, considered *a priori* confounders. For HIV prevalence at screening, age- and town-adjusted factors which reached p<0.05 were included in a multivariable model, and those with p>0.05 in the multivariable analysis were removed. Finally, all the omitted covariates were added back into the model and retained if p<0.05. The approach to investigate potential determinants of HSV-2 prevalence at enrolment, and HIV and HSV-2 incidence during follow-up, followed similar principles, but within a three-level conceptual framework since more-detailed data were collected at enrolment and follow-up, compared to screening. Firstly, age- and town-adjusted socio-demographic factors which reached p<0.05 were included in a multivariable model; those remaining independently associated at p<0.05 were retained in a core model. Secondly, behavioural factors were added to this core model one by one; those that were associated with the outcome at p<0.05, after adjusting for socio-demographic factors, were included in a multivariable model and retained if there remained evidence of an association (p<0.05). Thirdly, biological factors were assessed in a similar way. Lastly, all the omitted covariates were added back into the model and retained if p<0.05. For all final models, interactions were considered between covariates and town. Only variables which were measured in both the microbicides- and vaccines-preparedness studies were considered for inclusion in the final multivariable models. Individuals with missing data for a given variable were excluded from models which included that variable. However, for time-dependent variables, if results were not recorded at a given visit, either structurally (for infections not measured at every visit) or due to missingness, then the last observation was carried forward. Since some of the STIs are curable, we also conducted a sensitivity analysis assuming that *N. gonorrhoeae*, *C. trachomatis* and *T. vaginalis* were successfully treated and therefore were negative at subsequent visits, unless there were results to indicate otherwise.

## Results

### Screening and HIV Prevalence

Of 2,632 women screened, 669 (25%) were from Geita, 584 (22%) Kahama, 546 (21%) Shinyanga, and 833 (32%) Moshi. Their median age and duration of working in their current facility was 27 years (interquartile range, IQR: 22, 33) and 2 years (IQR: 1, 2), respectively. HIV status at screening was available for 2,229 (85%) women, of whom 358 were HIV-positive (16.1%; 95% confidence interval, CI: 14.6, 17.6). The characteristics of all women screened were broadly similar to those of the subset of women with HIV results.

### Factors Associated with HIV Prevalence at Screening

Most women (57%) with HIV results had completed primary (but not reached secondary) education, were Christians (74%), had one sexual partner in the last three months (62%) and were not cohabiting with a male partner (67%). A substantial proportion of women were separated or divorced or widowed (44%). In **Table 1** we present the associations between HIV prevalence, socio-demographic characteristics, and reported sexual behaviour.

**Table 1:** HIV prevalence at screening and associations with socio-demographic and sexual behaviour characteristics, among women working in food and recreational facilities in northern Tanzania.

Factor	N (column %) [1]	HIV prevalence,row %	Town- and age-adjustedOR (95% CI) [2]	Adjusted OR(95% CI) [2], [3]
**Overall**	2229 (100%)	16.10%	–	–
**Town**			P = 0.04	**P = 0.12**
Geita	639 (29%)	13.50%	1 (reference)	**1 (reference)**
Kahama	506 (23%)	18.80%	1.44 (1.04,1.99)	**1.46 (1.05,2.04)**
Shinyanga	520 (23%)	18.30%	1.18 (0.85,1.64)	**1.33 (0.95,1.87)**
Moshi	564 (25%)	14.50%	0.92 (0.66,1.28)	**1.15 (0.80,1.66)**
**Type of job**			P<0.001	**P<0.001**
Waitress	1047 (47%)	19.60%	1 (reference)	**1 (reference)**
*Mamalishe*	312 (14%)	9.60%	0.32 (0.21,0.48)	**0.37 (0.24,0.56)**
Other staff	870 (39%)	14.10%	0.52 (0.40,0.67)	**0.59 (0.45,0.77)**
**Age, years**			P<0.001	**P<0.001**
<20	200 (9%)	7.00%	1 (reference)	**1 (reference)**
20–24	676 (30%)	8.90%	1.30 (0.71,2.38)	**1.23 (0.66,2.29)**
25–29	539 (24%)	18.70%	3.06 (1.70,5.50)	**3.06 (1.65,5.68)**
≥30	814 (37%)	22.50%	3.94 (2.22,6.98)	**4.61 (2.47,8.60)**
**Highest formal education**			P<0.001	**P<0.001**
None/incomplete primary	608 (27%)	19.40%	1 (reference)	**1 (reference)**
Complete primary	1273 (57%)	16.90%	0.75 (0.58,0.98)	**0.73 (0.56,0.96)**
≥Secondary	347 (16%)	7.20%	0.33 (0.21,0.52)	**0.34 (0.21,0.54)**
**Religion**			P = 0.87	P = 0.88
Christian	1640 (74%)	16.20%	1 (reference)	1 (reference)
Muslim	573 (26%)	15.50%	0.95 (0.72,1.23)	0.95 (0.72,1.25)
None	13 (1%)	15.40%	1.29 (0.27,6.16)	0.75 (0.16,3.59)
**Marital status**			P<0.001	**P<0.001**
Married	507 (23%)	10.30%	1 (reference)	**1 (reference)**
Separated/divorced/widowed	985 (44%)	22.50%	2.76 (1.98,3.86)	**2.20 (1.56,3.10)**
Single	736 (33%)	11.40%	2.01 (1.34,2.99)	**1.78 (1.18,2.69)**
**N sex partners in last 3 months**			P = 0.004	P = 0.19
0	119 (5%)	14.30%	1 (reference)	1 (reference)
1	1362 (62%)	15.60%	1.21 (0.70,2.10)	1.31 (0.74,2.31)
2	380 (17%)	14.70%	1.30 (0.71,2.37)	1.06 (0.57,1.97)
≥3	328 (15%)	21.30%	2.20 (1.21,4.01)	1.59 (0.85,2.98)

OR = odds ratio, CI = confidence interval. Includes only those women whose HIV status is known at screening (85% of cohort). [1] Numbers may not add up to total due to missing data. [2] P-values from likelihood ratio test. The ORs for town are adjusted for age, and the ORs for age are adjusted for town. [3] Estimated ORs adjusted by town, age, type of job, education and marital status (the adjusted results for these variables are shown in bold).

In our final model, age, education, marital status, and type of job were independently associated with HIV. Although the point estimates indicated a slightly higher HIV prevalence in Kahama (18.8%), Shinyanga (18.3%), and Moshi (14.5%) compared with Geita (13.5%), there was no evidence of a difference overall (p = 0.12). Compared with women aged <20 years, HIV prevalence was significantly higher among those aged 25–29 (aOR = 3.06; 95% CI: 1.65, 5.68) and ≥30 years (aOR = 4.61; 95% CI: 2.47, 8.60). The prevalence of HIV was also higher in separated, divorced, or widowed (aOR = 2.20; 95% CI: 1.56, 3.10) and in single women (aOR = 1.78; 95% CI: 1.18, 2.69) compared with married women. HIV prevalence was lower among those working as *mamalishe* (aOR = 0.37; 95% CI: 0.24, 0.56) and other employment (aOR = 0.59; 95% CI: 0.45, 0.77) compared with waitresses, and among women who had completed primary (aOR = 0.73; 95%CI: 0.56, 0.96) or reached secondary education (aOR = 0.34; 95% CI: 0.21, 0.54) compared with those with incomplete primary education. There was no evidence of interactions between town and job type (p = 0.42), education level (p = 0.71) or marital status (p = 0.38).

### Enrolment and HSV-2 Prevalence

**Figure 1:**
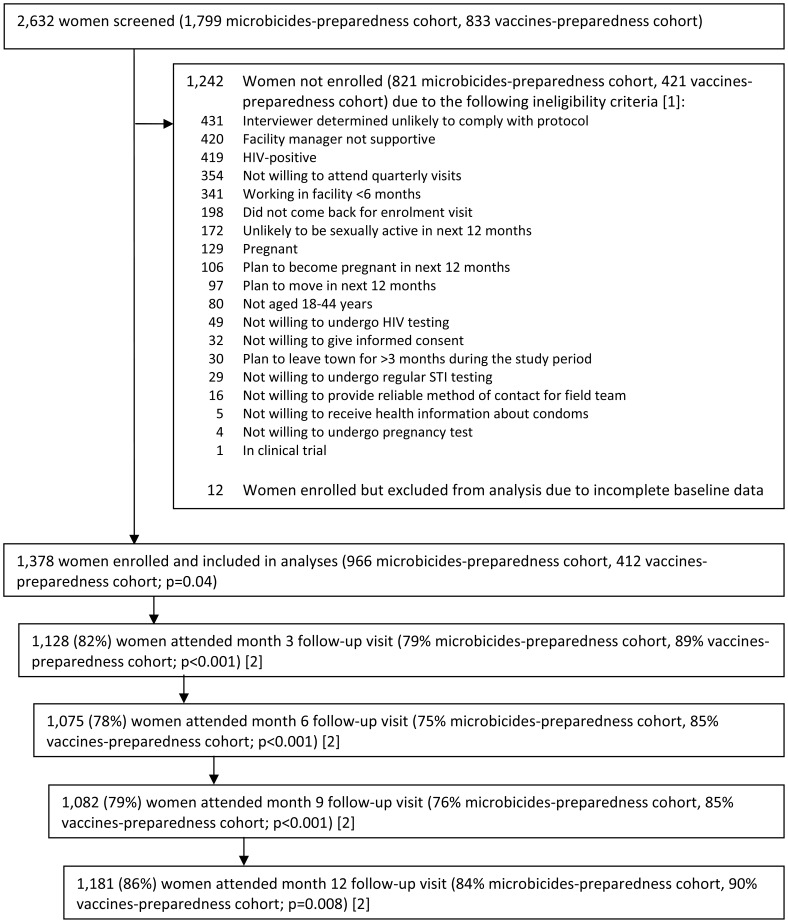
Screening, enrolment and follow-up of two cohorts of women working in food and recreational facilities in northern Tanzania. P-values are for the comparison in enrolment (of those screened) or attendance (of those enrolled) between the cohorts, by Χ^2^ test. [1] Some women met more than one ineligibility criteria; all reasons given are reported, therefore the sum is greater than the number of women not enrolled. [2] A window of +/−1.5 months was allowed for each visit, except the month 12 visit where no upper bound was applied (maximum 23 months). Restricting the upper limit of the month 12 visit to 13.5, 15 or 18 months yielded attendance of 81%, 84% and 86%, respectively.

Of those screened, 1,242 (47%) women were not enrolled (**Figure 1**). Women may have met >1 ineligibility criterion, with the most common reasons being unlikely to comply with the protocol (n = 431), lack of support from the facility manager (n = 420), being HIV-positive (n = 419), not willing to attend follow-up visits (n = 354) and working in the facility for less than 6 months (n = 341). Among 1,390 women enrolled, 12 were excluded from this analysis due to incomplete baseline data. Thus, 1,378 (966 (54% of those screened) microbicides-preparedness cohort and 412 (49%) vaccines-preparedness cohort; p = 0.04) women were included in this analysis. The characteristics of those enrolled were similar to those screened for the study. At enrolment, HSV-2 status was known for all but two women; 928 (67%) were HSV-2 positive (95% CI: 65, 70).

### Factors Associated with HSV-2 Prevalence at Enrolment

In **Table 2**, we present the associations between socio-demographic characteristics, sexual behaviour, biological factors and HSV-2 prevalence at enrolment. In our final model, we identified a number of independent predictors of HSV-2 prevalence. HSV-2 prevalence was lower in Moshi (52%, aOR = 0.45; 95% CI: 0.29, 0.71) and similar in other towns (Kahama, 75%; Shinyanga, 76%) compared with Geita (73%). HSV-2 prevalence was lower among women with secondary education or higher (46%, aOR = 0.53; 95% CI: 0.31, 0.88) compared with those with incomplete primary education (78%); and among women in other jobs besides *mamalishe* (64%, aOR = 0.67; 95% CI: 0.47, 0.95) compared with waitresses (67%).

**Table 2:** HSV-2 seroprevalence at enrolment and associations with socio-demographic, sexual behaviour and biological characteristics, among women working in food and recreational facilities in northern Tanzania.

Factor	N (column %) [1]	HSV-2 prevalence,row %	Town- and age-adjustedOR (95% CI) [2]	Adjusted OR(95% CI) [2], [3]
**Overall**	1376 (100%)	67%	–	–
**SOCIO-DEMOGRAPHIC FACTORS**				
**Town**			P<0.001	**P<0.001**
Geita	375 (27%)	73%	1 (reference)	**1 (reference)**
Kahama	305 (22%)	75%	1.14 (0.79,1.64)	**1.01 (0.62,1.66)**
Shinyanga	284 (21%)	76%	0.91 (0.62,1.32)	**0.98 (0.59,1.62)**
Moshi	412 (30%)	52%	0.29 (0.21,0.40)	**0.45 (0.29,0.71)**
**Type of job**			P = 0.02	**P = 0.02**
Waitress	608 (44%)	67%	1 (reference)	**1 (reference)**
*Mamalishe*	204 (15%)	78%	1.02 (0.68,1.54)	**1.20 (0.70,2.06)**
Other staff	564 (41%)	64%	0.69 (0.53,0.91)	**0.67 (0.47,0.95)**
**Age, years**			P<0.001	**P<0.001**
<20	105 (8%)	40%	1 (reference)	**1 (reference)**
20–24	431 (31%)	54%	2.17 (1.39,3.40)	**2.22 (1.18,4.18)**
25–29	344 (25%)	72%	4.92 (3.06,7.91)	**4.75 (2.44,9.26)**
≥30	496 (36%)	82%	9.86 (6.11,15.9)	**8.73 (4.37,17.5)**
**Highest formal education**			P<0.001	**P = 0.005**
None/incomplete primary	337 (25%)	78%	1 (reference)	**1 (reference)**
Complete primary	810 (59%)	69%	0.65 (0.47,0.90)	**1.04 (0.69,1.58)**
≥Secondary	228 (17%)	46%	0.33 (0.22,0.49)	**0.53 (0.31,0.88)**
**Marital status**			P<0.001	P = 0.32
Married	356 (26%)	69%	1 (reference)	1 (reference)
Separated/divorced/widowed	579 (42%)	79%	1.62 (1.17,2.25)	1.29 (0.84,1.98)
Single	441 (32%)	51%	0.90 (0.64,1.28)	1.42 (0.88,2.29)
**BEHAVIOURAL FACTORS**				
**CAGE score ** [**4**]			P<0.001	**P = 0.005**
Non-drinker or no problem drinking	873 (76%)	65%	1 (reference)	**1 (reference)**
Possible problem drinking	184 (16%)	76%	2.05 (1.38,3.05)	**2.06 (1.30,3.27)**
Probable problem drinking	95 (8%)	75%	1.72 (1.01,2.93)	**0.93 (0.49,1.79)**
**AUDIT score ** [**5**]			P = 0.003	P = 0.91
Non-drinker or low-risk	1184 (86%)	66%	1 (reference)	1 (reference)
Harmful or hazardous drinking	185 (14%)	77%	1.77 (1.20,2.62)	1.04 (0.57,1.87)
**Age at first sex, years**			P<0.001	**P = 0.02**
<16	361 (28%)	74%	1 (reference)	**1 (reference)**
≥16	915 (72%)	65%	0.60 (0.45,0.81)	**0.64 (0.44,0.95)**
**N lifetime sex partners**			P<0.001	**P = 0.02**
0–4	713 (61%)	59%	1 (reference)	**1 (reference)**
5–9	262 (23%)	72%	1.56 (1.11,2.20)	**1.12 (0.75,1.67)**
≥10	189 (16%)	83%	2.70 (1.73,4.23)	**2.13 (1.23,3.67)**
**N sex partners in last 12 months**			P = 0.005	P = 0.33
0–1	653 (50%)	64%	1 (reference)	1 (reference)
2	344 (27%)	70%	1.52 (1.10,2.09)	1.18 (0.77,1.80)
≥3	301 (23%)	73%	1.66 (1.16,2.37)	0.79 (0.44,1.40)
**N sex partners in last 3 months**			P = 0.007	P = 0.22
0	81 (6%)	63%	1 (reference)	1 (reference)
1	981 (72%)	66%	1.80 (1.06,3.05)	1.91 (0.97,3.77)
2	195 (14%)	74%	2.59 (1.40,4.78)	1.91 (0.83,4.39)
≥3	104 (8%)	76%	2.82 (1.40,5.68)	1.33 (0.51,3.46)
**Transactional sex in last year**			P = 0.05	P = 0.28
No	897 (65%)	65%	1 (reference)	1 (reference)
Yes	473 (35%)	73%	1.34 (1.00,1.80)	0.79 (0.51,1.21)
**Contraception**			P = 0.03	P = 0.12
None of the following	526 (38%)	71%	1 (reference)	1 (reference)
Condoms only	433 (32%)	59%	0.75 (0.55,1.01)	0.66 (0.45,0.99)
Pill (+/− condoms)	137 (10%)	74%	1.15 (0.73,1.80)	0.69 (0.39,1.21)
Injectable (DMPA; +/− condoms)	221 (16%)	72%	1.20 (0.82,1.75)	1.15 (0.71,1.86)
Other hormonal contraceptives	56 (4%)	66%	0.97 (0.51,1.84)	0.70 (0.33,1.49)
**BIOLOGICAL FACTORS**				
**Ever pregnant**			P<0.001	**P = 0.001**
No	208 (15%)	40%	1 (reference)	**1 (reference)**
Yes	1167 (85%)	72%	2.33 (1.63,3.33)	**2.14 (1.35,3.40)**
**Syphilis ** [**6**]			P<0.001	P<0.001
Negative	792 (82%)	71%	1 (reference)	1 (reference)
Previous infection	85 (9%)	95%	5.33 (1.90,15.0)	4.32 (1.26,14.9)
Active infection, low titre	41 (4%)	98%	16.1 (2.17,119)	12.5 (1.49,105)
Active infection, high titre	46 (5%)	76%	1.38 (0.66,2.86)	0.83 (0.28,2.44)
***T. vaginalis*** **** [**6**]			P = 0.20	P = 0.97
Negative	759 (80%)	74%	1 (reference)	1 (reference)
Positive	184 (20%)	76%	1.29 (0.87,1.92)	1.01 (0.59,1.73)
***C. trachomatis***			P = 0.92	P = 0.67
Negative	1185 (88%)	68%	1 (reference)	1 (reference)
Positive	157 (12%)	62%	0.98 (0.67,1.43)	0.90 (0.56,1.45)
***N. gonorrhoeae***			P = 0.11	P = 0.83
Negative	1290 (96%)	67%	1 (reference)	1 (reference)
Positive	51 (4%)	75%	1.71 (0.87,3.39)	1.10 (0.48,2.50)
**Bacterial vaginosis ** [**6**]			P = 0.005	P = 0.26
Negative	357 (37%)	71%	1 (reference)	1 (reference)
Indeterminate	160 (17%)	77%	1.69 (1.07,2.67)	1.37 (0.74,2.53)
Positive	446 (46%)	76%	1.69 (1.20,2.37)	1.47 (0.92,2.34)
**Genital ulcers on examination**			P = 0.003	**P = 0.01**
Negative	1249 (94%)	67%	1 (reference)	**1 (reference)**
Positive	84 (6%)	82%	2.39 (1.30,4.40)	**2.50 (1.15,5.40)**

OR = odds ratio, CI = confidence interval. Includes only those women whose HSV-2 status is known at enrolment (all but two). [1] Numbers may not add up to total due to missing data. [2] P-values from likelihood ratio test. The ORs for town are adjusted for age, and the ORs for age are adjusted for town. [3] Estimated ORs adjusted by town, age, job, education, CAGE category, age at first sex, number of partners in lifetime, genital ulcers on examination and ever pregnant (the adjusted results for these variables are shown in bold). While there was strong evidence of an association between HSV-2 prevalence and syphilis, this factor was not included in the fully-adjusted model, since it was only measured in the microbicides- and not the vaccines-preparedness cohort. [4] Based on responses to four CAGE questions. Score based on the number of positive answers: 0–1 = non-drinker or no problem drinking, 2 = possible problem drinking, ≥3 = probable problem drinking. [5] Based on responses to 10 AUDIT questions. Scores based on responses to each question: 0–7 non-drinker or low-risk, ≥8 harmful or hazardous drinking. [6] Results for syphilis, *T. vaginalis* (diagnosed by 72-hour culture) and bacterial vaginosis (diagnosed by Nugent criteria) not available for vaccines-preparedness cohort.

HSV-2 prevalence increased from 40% among women aged <20 years to 82% among those aged ≥30 years (aOR = 8.73; 95% CI: 4.37, 17.5, comparing those aged ≥30 with <20 years). Compared to women with no problem drinking (65%) as determined by the CAGE questionnaire, the HSV-2 prevalence was higher among those with possible problem drinking (76%, aOR = 2.06; 95% CI: 1.30, 3.27), although there was no evidence of a difference for those with probable problem drinking (75%, aOR = 0.93; 95%CI: 0.49, 1.79), perhaps due to small numbers of women in this latter category. Women who initiated sexual activity aged ≥16 years had lower HSV-2 prevalence (65%, aOR = 0.64; 95% CI: 0.44, 0.95) compared with those who initiated aged <16 years (74%). Other factors independently associated with higher HSV-2 prevalence were higher number of lifetime sexual partners, genital ulcers on examination, and having ever been pregnant. There was no evidence of interactions between town and any of these variables. Compared to those never infected, we observed higher prevalence of HSV-2 among those with previous (95%, aOR = 4.32; 95% CI: 1.26, 14.9) or low-titre active syphilis infection (98%, aOR = 12.5; 95% CI: 1.49, 105), although there was no evidence of a difference for those with high-titre active infection (76%, aOR = 0.83; 95% CI: 0.28, 2.44). Of note, these results are from the microbicides-preparedness cohort only.

### Follow-up and HIV and HSV-2 Incidence

Among 1,378 HIV-negative women in this analysis, attendance at 3, 6, 9 and 12 month visits was 82%, 78%, 79% and 86%, respectively (**Figure 1**). There were some differences in the attendance between the microbicides- and vaccines-preparedness cohorts, with 84% compared to 90%, respectively, attending the 12-month follow-up visit (p = 0.008). During the study period, with over 1200 PYRs of follow-up, there were 44 HIV seroconversions, yielding an overall HIV incidence of 3.7/100 PYRs (95% CI: 2.8, 5.1; **Figure S1**). Among 450 HSV-2-negative women and 339 PYRs of follow-up, we identified 97 HSV-2 seroconversions, resulting in an overall HSV-2 incidence of 28.6/100 PYRs (95% CI: 23.5, 35.0; **Figure S1**).

### Factors Associated with HIV Acquisition during Follow-up

HIV incidence ranged from 2.4/100 PYRs in Shinyanga to 3.8/100 PYRs in Moshi and 4.3/100 PYRs in Geita and Kahama, although there was no evidence of a difference by town (adjusted p = 0.47; **Table 3**). While HIV incidence was higher among younger women, there was no evidence of an age effect overall (adjusted p = 0.52). In our final model, adjusted for town and age, marital status and number of partners in last three months were independently associated with HIV incidence. Women who were separated, divorced, or widowed (adjusted incidence rate ratio (aRR) = 6.63; 95% CI: 1.97, 22.2) and single women (aRR = 2.93; 95% CI: 0.76, 11.3) were at higher HIV risk compared with those married. Similarly, women reporting two partners (aRR = 1.28; 95% CI: 0.48, 3.38) and ≥3 partners (aRR = 4.75; 95% CI: 2.10, 10.8) in the past three months were at higher risk compared with women with one or no partner. Due to low incidence, it was not possible to reliably estimate interactions of these variables with town. Of note, there was no evidence of an association between HSV-2 status and HIV incidence (p = 0.33). The pre-specified sensitivity analyses yielded point estimates substantially greater than one, but the confidence intervals were large and therefore the overall interpretations did not change (**Table S1**).

**Table 3:** HIV and HSV-2 incidence and associations with socio-demographic, behavioural and biological factors, among women working in food and recreational facilities in northern Tanzania.

	HIV			HSV-2 [1]		
Factor	Cases/person-years (rate per 100 person-years) [1]	Town- and age-adjusted RR (95% CI) [2]	Adjusted RR (95% CI) [2], [3]	Cases/person-years (rate per 100 person-years) [1]	Town- and age-adjusted RR (95% CI) [2]	Adjusted RR (95% CI) [2], [4]
**Overall**	44/1200 (3.7)	–	–	97/339 (28.6)		
**SOCIO-DEMOGRAPHIC FACTORS AT ENROLMENT**						
**Town**		P = 0.62	**P = 0.47**		P = 0.39	**P = 0.07**
Geita	13/305 (4.3)	1 (reference)	**1 (reference)**	20/69 (28.9)	1 (reference)	**1 (reference)**
Kahama	11/257 (4.3)	1.03 (0.46,2.30)	**0.95 (0.42,2.15)**	16/61 (26.1)	0.97 (0.50,1.88)	**1.09 (0.56,2.13)**
Shinyanga	6/249 (2.4)	0.57 (0.21,1.50)	**0.68 (0.25,1.83)**	11/55 (20.1)	0.70 (0.33,1.47)	**0.69 (0.32,1.47)**
Moshi	14/370 (3.8)	0.90 (0.42,1.92)	**1.43 (0.65,3.17)**	50/153 (32.6)	1.21 (0.71,2.06)	**1.55 (0.89,2.69)**
**Type of job**		P = 0.10	P = 0.46		P = 0.92	P = 0.86
Waitress	24/501 (4.8)	1 (reference)	1 (reference)	41/150 (27.4)	1 (reference)	1 (reference)
*Mamalishe*	3/185 (1.6)	0.32 (0.09,1.07)	0.49 (0.14,1.70)	9/35 (25.7)	1.01 (0.47,2.19)	0.95 (0.43,2.10)
Other staff	17/494 (3.4)	0.70 (0.37,1.33)	0.97 (0.50,1.88)	47/154 (30.5)	1.09 (0.71,1.68)	1.11 (0.72,1.73)
**Age, years**		P = 0.79	**P = 0.52**		P = 0.45	**P = 0.35**
<20	4/74 (5.4)	1.28 (0.43,3.81)	**1.75 (0.53,5.76)**	14/40 (35.2)	1.17 (0.59,2.29)	**1.34 (0.63,2.88)**
20–24	13/353 (3.7)	0.87 (0.42,1.79)	**0.94 (0.43,2.06)**	36/149 (24.2)	0.73 (0.44,1.23)	**0.76 (0.43,1.32)**
25–29	9/298 (3.0)	0.73 (0.33,1.63)	**0.69 (0.30,1.56)**	22/74 (29.9)	0.92 (0.52,1.65)	**0.83 (0.46,1.51)**
≥30	18/455 (4.0)	1 (reference)	**1 (reference)**	25/76 (32.8)	1 (reference)	**1 (reference)**
**Highest formal education**		P = 0.55	P = 0.55		P = 0.01	**P = 0.01**
None/incomplete primary	10/276 (3.6)	1 (reference)	1 (reference)	23/49 (46.5)	1 (reference)	**1 (reference)**
Complete primary	29/707 (4.1)	1.17 (0.56,2.45)	1.40 (0.65,3.00)	54/192 (28.1)	0.55 (0.33,0.92)	**0.56 (0.33,0.92)**
≥Secondary	5/197 (2.5)	0.71 (0.23,2.14)	0.96 (0.31,2.98)	19/96 (19.7)	0.38 (0.20,0.72)	**0.38 (0.20,0.72)**
**Marital status**		P<0.001	**P<0.001**		P = 0.05	**P = 0.04**
Married	3/325 (0.9)	1 (reference)	**1 (reference)**	22/90 (24.3)	1 (reference)	**1 (reference)**
Separated/divorced/widowed	30/492 (6.1)	7.25 (2.17,24.2)	**6.63 (1.97,22.2)**	35/87 (40.1)	1.82 (1.05,3.16)	**1.97 (1.13,3.42)**
Single	11/363 (3.0)	3.05 (0.79,11.7)	**2.93 (0.76,11.3)**	40/161 (24.9)	1.08 (0.60,1.95)	**1.24 (0.68,2.25)**
**BEHAVIOURAL FACTORS AT ENROLMENT**						
**Age at first sex, years**		P = 0.75	P = 0.96		P = 0.19	P = 0.49
<16	13/310 (4.2)	1 (reference)	1 (reference)	23/66 (34.9)	1 (reference)	1 (reference)
≥16	29/784 (3.7)	0.89 (0.46,1.74)	1.02 (0.52,2.01)	66/249 (26.5)	0.72 (0.44,1.16)	0.84 (0.51,1.38)
**TIME-VARYING BEHAVIOURAL FACTORS**						
**N sex partners in last 3 months**		P = 0.001	**P = 0.005**		P = 0.21	P = 0.23
0–1	31/993 (3.1)	1 (reference)	**1 (reference)**	85/300 (28.3)	1 (reference)	1 (reference)
2	5/128 (3.9)	1.45 (0.55,3.82)	**1.28 (0.48,3.38)**	10/25 (40.0)	1.82 (0.93,3.57)	1.66 (0.84,3.26)
≥3	8/56 (14.4)	6.04 (2.67,13.7)	**4.75 (2.10,10.8)**	2/13 (15.7)	0.68 (0.16,2.79)	0.55 (0.13,2.27)
**Transactional sex in past 3 m**		P = 0.06	P = 0.83		P = 0.42	P = 0.73
No	33/969 (3.4)	1 (reference)	1 (reference)	85/291 (29.2)	1 (reference)	1 (reference)
Yes	11/209 (5.3)	2.08 (1.01,4.30)	1.10 (0.47,2.59)	12/46 (26.1)	1.31 (0.69,2.46)	1.12 (0.59,2.13)
**Contraception**		P = 0.28	P = 0.24		P = 0.77	P = 0.82
None of the following	18/492 (3.7)	1 (reference)	1 (reference)	40/134 (29.8)	1 (reference)	1 (reference)
Condoms only	11/350 (3.1)	0.90 (0.42,1.91)	0.70 (0.32,1.51)	28/119 (23.6)	0.88 (0.54,1.44)	0.91 (0.55,1.50)
Pill (+/− condoms)	4/115 (3.5)	0.75 (0.25,2.24)	0.68 (0.23,2.04)	9/26 (34.6)	1.03 (0.53,2.00)	1.11 (0.56,2.17)
Injectable (DMPA; +/− condoms)	11/183 (6.0)	1.89 (0.88,4.08)	1.63 (0.75,3.52)	17/48 (35.1)	1.25 (0.69,2.24)	1.24 (0.68,2.25)
Other hormonal contraceptives	0/40 (0.0)	[5]	[5]	3/11 (28.5)	[5]	[5]
**TIME-VARYING BIOLOGICAL FACTORS**						
**HSV-2 status**		P = 0.15	P = 0.33			
Negative	8/349 (2.3)	1 (reference)	1 (reference)	–	–	–
Positive (from baseline)	34/804 (4.2)	2.13 (0.94,4.81)	1.79 (0.77,4.12)			
Positive (seroconverted during follow-up)	1/26 (3.9)	1.11 (0.14,8.96)	0.95 (0.12,7.72)			
**Syphilis ** [**6**]		P = 0.83	P = 0.57		P = 0.24	P = 0.16
Negative	26/661 (3.9)	1 (reference)	1 (reference)	43/172 (25.0)	1 (reference)	1 (reference)
Previous infection	2/77 (2.6)	0.73 (0.17,3.16)	0.72 (0.16,3.15)	3/5 (61.7)	2.49 (0.75,8.24)	1.59 (0.45,5.61)
Active infection	2/73 (2.7)	0.72 (0.17,3.06)	0.51 (0.12,2.20)	1/9 (11.4)	0.43 (0.06,3.13)	0.24 (0.03,1.85)
***T. vaginalis*** ** infection ** [**6**]		P = 0.58	P = 0.80		P = 0.30	P = 0.50
Negative	24/661 (3.6)	1 (reference)	1 (reference)	35/150 (23.4)	1 (reference)	1 (reference)
Positive	6/138 (4.4)	1.30 (0.53,3.19)	1.13 (0.46,2.78)	9/32 (28.1)	1.50 (0.71,3.17)	1.31 (0.61,2.85)
***C. trachomatis*** ** infection**		P = 0.87	P = 0.71		P = 0.27	P = 0.52
Negative	41/1100 (3.9)	1 (reference)	1 (reference)	85/303 (28.1)	1 (reference)	1 (reference)
Positive	3/105 (2.9)	0.90 (0.28,2.96)	0.81 (0.24,2.65)	10/32 (30.9)	1.48 (0.76,2.87)	1.26 (0.64,2.50)
***N. gonorrhoeae*** ** infection**		P = 0.37	P = 0.65		P = 0.86	P = 0.67
Negative	41/1100 (3.7)	1 (reference)	1 (reference)	92/325 (28.3)	1 (reference)	1 (reference)
Positive	3/44 (6.8)	1.80 (0.55,5.88)	1.33 (0.40,4.37)	3/10 (28.9)	0.90 (0.28,2.89)	0.79 (0.24,2.52)
**Bacterial vaginosis infection ** [**6**]		P = 0.32	P = 0.34		P = 0.24	P = 0.25
Negative	10/305 (3.3)	1 (reference)	1 (reference)	14/81 (17.4)	1 (reference)	1 (reference)
Indeterminate	3/149 (2.0)	0.58 (0.16,2.10)	0.48 (0.13,1.78)	10/32 (31.6)	1.76 (0.78,4.00)	1.60 (0.68,3.76)
Positive	17/357 (4.8)	1.34 (0.61,2.95)	1.11 (0.50,2.48)	23/73 (31.4)	1.68 (0.85,3.31)	1.74 (0.88,3.45)
**Genital ulcers on examination**		P = 0.22	P = 0.29		P = 0.69	P = 0.75
Negative	40/1100 (3.6)	1 (reference)	1 (reference)	91/325 (28.0)	1 (reference)	1 (reference)
Positive	4/58 (6.9)	2.03 (0.72,5.74)	1.85 (0.65,5.26)	3/9 (31.9)	1.28 (0.40,4.08)	1.21 (0.38,3.89)
**HIV**					P = 0.42	P = 0.37
Negative	–	–	–	95/336 (28.3)	1 (reference)	1 (reference)
Positive				2/2 (81.0)	1.92 (0.46,8.11)	2.07 (0.49,8.74)

RR = incidence rate ratio, CI = confidence interval. [1] Numbers may not add up to total due to missing data. [2] P-values from likelihood ratio test. The ORs for town are adjusted for age, and the ORs for age are adjusted for town. [3] Estimated RRs adjusted for town, age, marital status and time-updated number of partners in last 3 months (the adjusted results for these variables are shown in bold). [4] Estimated RRs adjusted for town, age, education and marital status (the adjusted results for these variables are shown in bold). [5] Other hormonal contraceptives grouped with pill, due to low numbers. [6] Results for syphilis, *T. vaginalis* (diagnosed by 72-hour culture) and bacterial vaginosis (diagnosed by Nugent criteria) not available for vaccines-preparedness cohort.

### Factors Associated with HSV-2 Acquisition during Follow-up

HSV-2 incidence ranged from 20.1/100 PYRs in Shinyanga to 32.6/100 PYRs in Moshi (**Table 3**). In our final model, there was some evidence to suggest a difference by town, with those in Moshi at highest HSV-2 risk, compared with Geita (aRR = 1.55; 95% CI: 0.89, 2.69; global p = 0.07). Adjusted for town and age, education and marital status were independently associated with HSV-2 incidence. Compared with women with incomplete primary education, those who had completed primary education (aRR = 0.56; 95% CI: 0.33, 0.92) or reached secondary education or above (aRR = 0.38; 95% CI: 0.20, 0.72) were at lower HSV-2 risk. Women who were separated, divorced, or widowed (aRR = 1.97; 95% CI: 1.13, 3.42) and single women (aRR = 1.24; 95% CI: 0.68, 2.25) were at higher HSV-2 risk compared with married women.

## Discussion

In this study of women considered to be at increased risk of HIV and other STIs in northern Tanzania, the prevalences of HIV and HSV-2 were indeed high, at 16% and 67% respectively, indicating that these infections continue to pose a major public health problem in this population. While the prevalence of HIV was somewhat lower than in earlier studies conducted in similar cohorts [5], [7], [15]–[17], it remained higher than that reported among women in the general population [3]. Women working in these settings are known to be involved in complex sexual networks including multiple partners and exchange of sex for gifts or money [15], [17]. The prevalence of HSV-2 and other STIs was comparable to earlier studies conducted in these settings [5], [15], [17], [18]. Thus, scaling-up of known effective interventions against HIV and other STIs is a major priority in this population.

As expected, HIV and HSV-2 prevalence shared a number of common risk factors including age, marital status, job type and level of education. As observed in other studies [15], [17]–[19], the prevalence of HIV and HSV-2 infections was higher in older participants, due to age being a proxy measure of the duration of sexual activity and these being life-long infections. Women who were separated, divorced, or widowed had the highest prevalence of both infections. Such women may have been exposed to HIV in their previous marriages and/or may engage with multiple sex partners due to relative personal freedom and as they attempt to establish new relationships. We also observed higher risk of HIV and HSV-2 among waitresses compared with other type of jobs, and lower risk associated with higher level of education. Level of education has been associated with increased awareness about STIs and use of protective devices such as condoms [20], although we did not observe this in our study (data not shown). The observed associations suggest that structural factors, such as the level of education, may be important drivers of the infections in this population.

Only limited data on potential behavioural risk factors were collected during the screening visit, constraining our ability to assess their associations with HIV prevalence. During enrolment, and subsequent follow-up visits, a substantial proportion of women reported high-risk sexual behaviours. Earlier sexual debut and higher number of lifetime sexual partners were associated with HSV-2 prevalence. HSV-2 infection was also associated with possible problem alcohol drinking based on the CAGE questionnaire. Alcohol consumption has been consistently related to risky sexual behaviours, including multiple sex partners and lack of condom use [21].

The HIV incidence observed in this study was comparable to similar cohorts in Moshi and Mwanza, Tanzania, and South Africa [4]–[6], [19], but lower than that observed in another similar cohort in Mbeya, Tanzania [22] and among women from the general population in South Africa [23], [24]. The HSV-2 incidence was higher than that observed in similar cohorts in Moshi and Mwanza [5], [25], but comparable to Kenyan women with high-risk sexual behaviour [26], [27] and women attending sexual health clinics in Alabama, US [28].

Both infections showed a trend toward increased acquisition among women aged below 20, compared with over 30 years, although the evidence was weak. Other studies have noted this pattern of high HIV incidence among young women [4], [24], [29], underscoring the vulnerability of young women to HIV and other STIs. Similar patterns of associations for marital status and education were observed for incidence as for prevalence. Of the behavioural risk factors we examined, only the number of sex partners during the past three months played a role, with a higher number of partners independently associated with higher HIV incidence. Although our models suffered from low power, in general, associations with sexual behaviour variables were relatively stronger for HIV than for HSV-2 infection. We did not observe any associations between biological factors and either HIV or HSV-2 incidence; this may be due to limited power or perhaps curable STIs being diagnosed and treated and therefore no longer present at the time of HIV infection.

A strength of this study is the inclusion of women from four towns, across two cohort studies which were conducted in comparable ways. Although this is a highly-mobile population, we achieved a good retention rate of 86% over 12 months follow-up. We explored a range of potential risk factors, covering socio-demographic, sexual, and other behaviour and clinical variables, although self-reported behavioural factors are likely to suffer from social desirability and recall bias.

While the two cohort studies were conducted in very similar ways, there were some differences in the laboratory methods applied. HIV confirmatory testing algorithms and HSV-2 test kits differed between the two cohorts, and the two HSV-2 assays used are known to have different performance in African settings [30]. Syphilis results were not available for the vaccines-preparedness cohort. While bacterial vaginosis and *T. vaginalis* were diagnosed by Amsel clinical criteria [31] and wet mount, respectively, in the vaccines-preparedness cohort, we used only the results from the microbicides-preparedness cohort, where these infections were diagnosed by gold standard methods (Nugent criteria [32] and 72-hour culture [33], respectively). An implication of these differences is that we had reduced power to assess the associations of HIV and HSV-2 infection with factors that were only measured in one cohort. We also had limited power to investigate interactions of risk factors with town. Further, there was some evidence of better retention in the vaccines-preparedness cohort, perhaps due to this study team having worked in Moshi town with similar cohorts in a number of previous studies [6], [15], and/or greater mobility among the women enrolled in the microbicides-preparedness cohort (data not shown).

In summary, these results confirm that women working in hotels, bars and other food and recreational facilities in northern Tanzania continue to be at increased risk of both HIV and HSV-2 infections, and may be one of the core groups playing an important role in the continued transmission of HIV. Identification of core groups in countries such as Tanzania, where the HIV epidemic is generalised, is an important initial step before the design of targeted HIV prevention interventions. We identified a number of demographic characteristics, including age, marital status, level of education, and reported sexual behaviours, which were independently associated with increased risk of infections in this population. This knowledge may be used to identify women at highest risk of HIV infection for inclusion in future HIV prevention trials or to design interventions that address the individual and structural drivers of the epidemic. Development and testing of new promising interventions among the most affected populations in rigorously conducted clinical trials remain a major priority for Africa. Although recruiting and retaining participants at highest risk of HIV in clinical trials may be difficult [5], we have demonstrated the feasibility of undertaking such trials in highly-mobile populations, with the possibility of maintaining high retention rates during the follow-up period.

## Supporting Information

Figure S1 Kaplan-Meier plots of (a) HIV and (b) HSV-2 incidence by town.
(TIF)

Table S1 Sensitivity analysis: HIV incidence and associations with curable STIs.
(DOCX)
