# Supplementary figures and images for: The Epidemiology of HIV and HSV-2 Infections among Women Participating in Microbicide and Vaccine Feasibility Studies in Northern Tanzania
Source: PLoS One. 2013 Jul 18;8(7):e68825. doi: 10.1371/journal.pone.0068825 (PMC3715536; doi:10.1371/journal.pone.0068825)

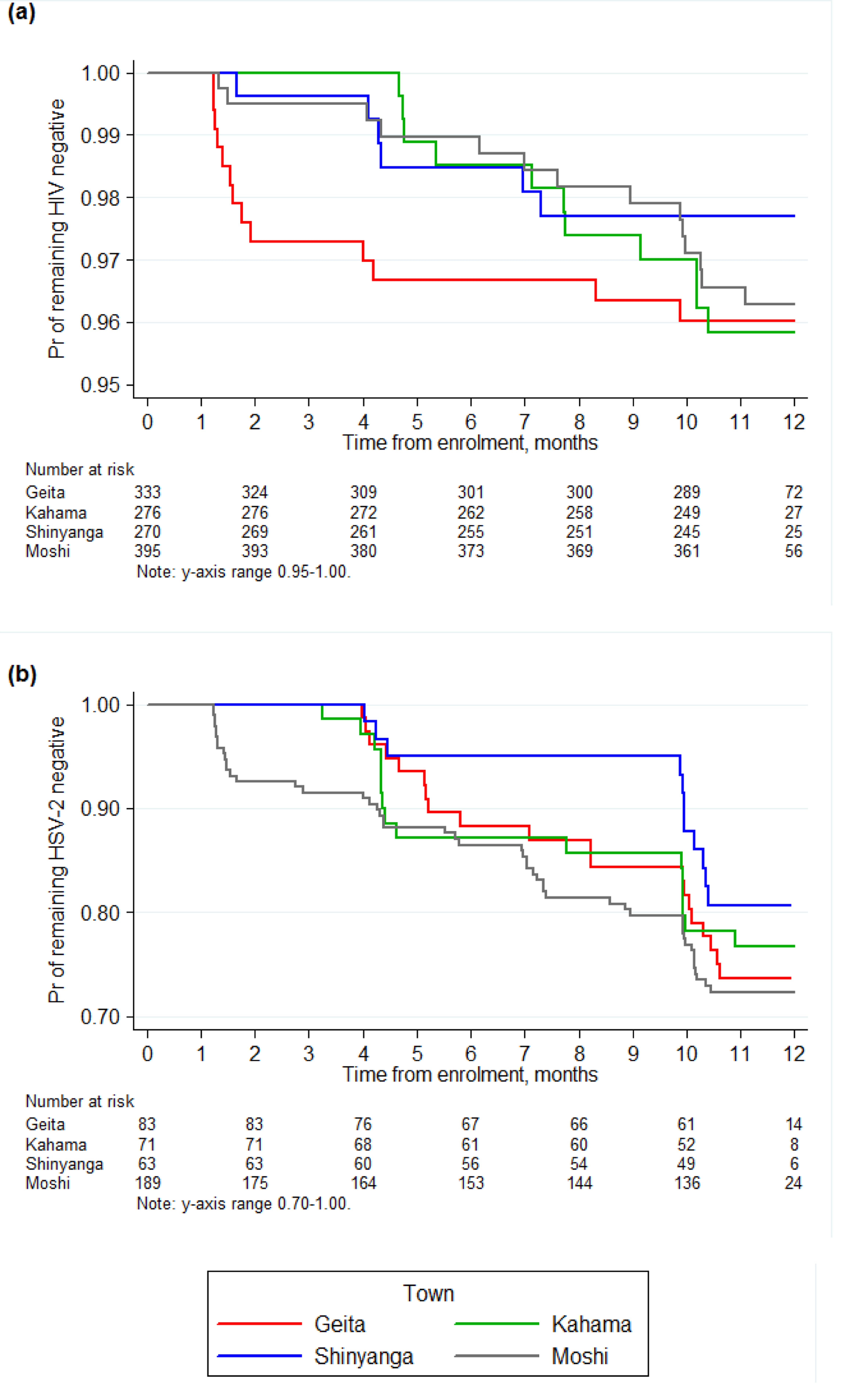

Supplement: Figure S1 — Kaplan-Meier plots of (a) HIV and (b) HSV-2 incidence by town. (TIF) [file pone.0068825.s001.tif]
