# Supplementary material for: The Epidemiology of HIV and HSV-2 Infections among Women Participating in Microbicide and Vaccine Feasibility Studies in Northern Tanzania
Source: PLoS One. 2013 Jul 18;8(7):e68825. doi: 10.1371/journal.pone.0068825 (PMC3715536; doi:10.1371/journal.pone.0068825)
Supplement: Table S1 — Sensitivity analysis: HIV incidence and associations with curable STIs. (DOCX) [file pone.0068825.s002.docx]

**Table S1. Sensitivity analysis: HIV incidence and associations with curable STIs.**

|  | **Original analysis [1]** | | **Sensitivity analysis [2]** | |
| --- | --- | --- | --- | --- |
|  | **Town- and age-adjusted RR (95% CI)** | **P-value [3]** | **Town- and age-adjusted RR (95% CI)** | **P-value [3]** |
| ***N. gonorrhoeae* infection** | 1.80 (0.55,5.88) | 0.37 | 2.60 (0.63,10.8) | 0.25 |
| ***C. trachomatis* infection** | 0.90 (0.28,2.96) | 0.87 | 1.78 (0.54,5.84) | 0.38 |
| ***T. vaginalis* infection [6]** | 1.30 (0.53,3.19) | 0.58 | 2.04 (0.71,5.85) | 0.23 |

[1] As shown in Table 3 of the main manuscript.

[2] Under the assumption that the curable STIs are treated and cured by the next visit (unless there are test results to indicate otherwise).

[3] Likelihood ratio test.
